# Bilateral temporomandibular joint dislocation in a 29-year-old man: a case report

**DOI:** 10.1186/1752-1947-4-263

**Published:** 2010-08-10

**Authors:** Tanujan Thangarajah, Neil Mcculloch, Suthan Thangarajah, Judith Stocker

**Affiliations:** 1 University Hospital Coventry and Warwickshire, Clifford Bridge Road, Coventry, CV2 2DX, UK; 2 University of Leicester Medical School, Maurice Shock Building, PO Box 138, University Road, Leicester, LE1 9HN, UK

## Abstract

**Introduction:**

A dislocation of the temporomandibular joint represents three percent of all reported dislocated joints.

The treatment entails reduction of the deformity and this can often be achieved in a ward setting.

**Case presentation:**

We present the case of a 29-year-old Caucasian man with a non-traumatic bilateral anterior temporomandibular joint dislocation. Following several unsuccessful attempts, due to both inadequate patient analgesia and sedation, joint reduction had to be performed in theatre with the patient under general anesthesia.

**Conclusion:**

This case highlights the importance of providing the patient with adequate analgesia and sedation when attempting the reduction of temporomandibular joint dislocations.

## Introduction

A dislocation of the temporomandibular joint (TMJ) represents three percent of all reported dislocated joints [1]. The majority of cases are non-traumatic and are often precipitated by yawning, eating, dental treatment, endoscopy, or oral intubation. The diagnosis is confirmed by radiography and immediate closed reduction should be performed [2]. Pivotal to achieving joint reduction is adequate patient analgesia and sedation where appropriate. We present the case of a man with non-traumatic bilateral anterior TMJ dislocation and discuss his management.

## Case presentation

A well-built 29-year-old Caucasian man attended the emergency department in the late evening unable to close his mouth immediately after yawning. Although he had no medical problems, he did admit to having long-standing 'clicking' of his left TMJ. He was not on any regular medication, was a non-smoker and drank alcohol socially. His occupation was as an electrician.

On examination his mouth was open and there was significant drooling. No oral or facial lacerations were present. Bilaterally, there was a depression in the his pre-auricular area associated with severe tenderness. Radiographs were taken and illustrated bilateral anterior dislocations of the patient's TMJs. A closed reduction was attempted in the emergency department using 5 mg midazolam and 5 mg diazepam; however, after two attempts reduction could not be achieved. At this point, the maxillofacial team in the hospital were contacted. He was subsequently transferred later that evening to a regional oral and maxillofacial surgical unit.

Upon further questioning, he admitted that during the previous attempts at joint reduction he had been in considerable pain and was unable to relax his muscles of mastication. With this in mind, a further reduction was attempted on a ward by a doctor specialising in oral and maxillofacial surgery. However, he was unable to relax his muscles sufficiently to facilitate reduction because of his ongoing anxiety and apprehension over the procedure. He also refused both local and regional anesthetic techniques due to the previous unsuccessful attempts and was insistent upon the use of general anesthesia. Given that this would occur only a few hours later in the morning, it was deemed a suitable option for him.

The patient was promptly taken to theatre in the morning and with the aid of a muscle relaxant, both of his TMJs were reduced easily by applying force in an inferior direction to the mandibular rami. To maintain position, a Philadelphia collar was applied for 24 hours and he was advised to avoid excessive jaw movements. Postoperative radiographs indicated that his mandibular condyles had been restored to their correct position within his glenoid fossae. He was discharged from hospital the following day with no further follow up as per institutional policy. Advice regarding the possibility of further dislocation and instability were given to him prior to discharge.

## Discussion

A dislocation of the TMJ is defined as the excessive forward movement of the mandibular condyle beyond the articular eminence with complete separation of the articular surfaces and fixation in that position [3]. Several morphological variants exist based on the position of the condyle in relation to the articular eminence; these are termed anterior, posterior, superior, and lateral [1]. However, the anterior subtype is most common [4].

Several precipitants have been identified for dislocation and can be classified as either traumatic or non-traumatic. Non-traumatic precipitants are more common and include laughing, taking a large bite of something, convulsions, and yawning [1,5]. In addition to these, predisposing factors to dislocation have also been recognized. These include poor joint capsule integrity, weak articular eminence morphology, and muscle hypotonicity [5].

The diagnosis of TMJ dislocation is often clinically based in the first instance. Typical signs and symptoms seen in patients with TMJ dislocation include mandibular pain, an inability to occlude the teeth, pre-auricular depressions, and a prominent mandibular head anteriorly (anterior variant) [6]. The recognition of any of the aforementioned features should prompt an immediate radiograph to confirm the diagnosis.

The treatment of a patient with TMJ dislocation entails reduction of the deformity, which can be accomplished in either a ward or the emergency department setting. It is imperative that this procedure is performed with little delay because complications such as fracture become increasingly apparent if time is wasted. Furthermore, spasms of both the masseter and pterygoid muscles worsens as time elapses, therefore making the reduction procedure more difficult [2].

To achieve reduction of an anterior dislocation, downward pressure should be applied on the patient's lower molar teeth while elevating the angle of the mandible with the fingers and pushing the entire mandible posteriorly. To prevent the patient from biting down on the examiners' hands, swabs can be applied over the patient's lower molars. The two major inhibiting factors for reduction are elevator muscular spasm and pain both in and around the joint capsule. These both increase if dislocation is prolonged and thus various methods to counter them have been proposed [7].

One commonly employed technique to counter the inhibiting factors for reduction is to ask the patient to open his or her mouth widely or against resistance. This reduces the tone of the patient's elevator muscles, thereby permitting reduction. Sedatives are also routinely used but when given intravenously require cardio-respiratory monitoring of the patient. Muscle relaxants have also been utilized; however, if given orally they may take up to one hour to take effect and there can be difficulty encountered in the patient swallowing the tablet. Some more novel techniques to relax the facial muscles include the infiltration of local anesthesia around the joint capsule and use of specific nerve blocks. Although use of local anesthetic is recognized to reduce pain, it does not have the same effect on muscle spasm. The use of nerve blocks has been extensively described in the literature with varying outcomes. Specific nerves that have been targeted include the mandibular, deep temporal and masseteric nerves. For example, mandibular nerve blocks have been traditionally used during a number of dental procedures and have more recently been used to provide analgesia for patients undergoing surgery in the vicinity of the mandibular region. In conjunction with sedation, it has been found to be a safe alternative to general anesthesia for patients undergoing closed reduction of TMJ dislocation [1,2,4,7].

Despite the success of such conservative measures, there remain a proportion of patients that require closed reduction under general anesthesia. However, when this fails other more invasive procedures such as temporal myotomy or sagittal split mandibular osteotomy may be used [7]. The post-reduction care typically involves dietary control, use of anti-inflammatory medications, immobilisation of the mandible and mouth-opening training [1]. After a short period of immobilisation, it is imperative that limited movement of the patient's jaw is commenced. For those patients who subsequently undergo recurrent dislocations, operative intervention is usually required [1].

In the current case several attempts were made to reduce the TMJ. In hindsight, however, it is clear that insufficient quantities of both analgesia and sedation were used given the patients' large build. This made reduction extremely difficult and deterred him from accepting alternative methods of anesthesia, such as a nerve block or local anesthetic infiltration. It is for this reason that general anesthesia of this patient was used.

## Conclusions

TMJ dislocation is easily managed and completely treatable. To achieve a satisfactory outcome both adequate analgesia and muscle relaxation should be ensured. The patients' weight should be considered because insufficient doses of analgesia may render the reduction unsuccessful. This may in turn make the patient extremely anxious and prevent him or her from accepting the other anesthetic techniques outlined above. However, closed reduction under general anesthesia, even when performed several hours after the initial dislocation, is still a relatively simple and effective procedure.

## Competing interests

The authors declare that they have no competing interests.

## Authors' contributions

TT conceived the study, assisted with treatment, acquired and interpreted the data, and wrote the manuscript. NM assisted with treatment, acquired and interpreted the data, and helped formulate the manuscript. ST conducted a literature search, acquired references, and helped formulate the manuscript. JS assisted with treatment, supervized the entire management of the patient, and critically reviewed the paper for its intellectual content. All authors have read and approved the final manuscript.

## Consent

Written informed consent was obtained from the patient for publication of this case report and any accompanying images. A copy of the written consent is available for review by the Editor-in-Chief of this journal.
